# Dental Fluoride Varnish Application During Medical Visits Among Children Who Are Privately Insured

**DOI:** 10.1001/jamanetworkopen.2021.22953

**Published:** 2021-08-30

**Authors:** Kimberley H. Geissler, Andrew W. Dick, Sarah L. Goff, Christopher Whaley, Ashley M. Kranz

**Affiliations:** 1 University of Massachusetts Amherst School of Public Health and Health Sciences, Amherst; 2 RAND, Boston, Massachusetts; 3 RAND, Santa Monica, California; 4 RAND, Arlington, Virginia

## Abstract

This cross-sectional study examines fluoride varnish application rates during well-child medical visits and identify characteristics associated with fluoride varnish receipt.

## Introduction

Fluoride varnish is effective at reducing tooth decay, which affects nearly a quarter of US children ages 2 to 5 years and more than half of children ages 6 to 8 years.^1,2^ To increase young children’s receipt of preventive oral health services, the US Preventive Services Task Force recommends medical providers apply fluoride varnish to young children’s teeth during well-child visits through 5 years of age.^2^ Offering fluoride varnish in medical settings may increase young children’s receipt of this service because 89% of children younger than 6 years of age had a preventive medical visit in 2019.^3^ However, fewer than 8% of young Medicaid-enrollees receive fluoride varnish in medical settings,^4^ and no studies have examined fluoride varnish applications during medical visits for children who are privately insured. Studying children who are privately insured is important because coverage of this service without cost-sharing has been mandated since 2015,^5^ and fewer than 1 in 3 children under 5 years who are privately insured have an annual dental visit.^6^ We used data from 4 states to examine fluoride varnish application rates during well-child medical visits and identify characteristics associated with fluoride varnish receipt.

## Methods

This cross-sectional study was approved by RAND’s institutional review board, and a waiver of informed consent was granted because of the use of deidentified patient data. This study followed the Strengthening the Reporting of Observational Studies in Epidemiology (STROBE) reporting guideline.

We used 2016 to 2018 data for children who are privately insured from all payer-claims databases from Maine, Connecticut, New Hampshire, and Rhode Island. We limited the analytic sample to children ages 1 to 5 years and identified well-child visits using CPT codes 99381-3 and 99391-3. Well-child visit periodicity schedules vary by age; thus, our unit of analysis was the well-child visit. We identified fluoride varnish applications (ie, CPT code 99188 and CDT code D1206) during well-child visits with the same service date.

We calculated descriptive statistics and estimated the unadjusted and adjusted odds of a visit including fluoride varnish using logistic regression; we then calculated regression-adjusted probabilities of fluoride application (eMethods in the Supplement). Tests were 2-tailed, statistical significance was set at *P* *<* .05, and we used county-level cluster robust standard errors. Data analyses were performed using SAS Version 9.4 and Stata-MP version 16.1 (StataCorp). Analysis was performed during November 2020 to March 2021.

## Results

The sample included 328 661 well-child visits for children aged 1 to 5 years (169 001 [51.4%] male; 132 563 [40.3%] were 1-year-olds) Of the visits, 134 662 (41.0%) occurred in Connecticut and 15 756 (4.8%) included fluoride varnish applications (Table). Fluoride varnish was more common among visits for younger children, as illustrated by unadjusted and regression-adjusted probabilities (Figure). A 2-year-old was 7.7 percentage points (pp) (95% CI, 5.9 pp-9.4 pp) more likely to receive fluoride varnish than a 5-year-old. From 2016 to 2018, the regression-adjusted probability of fluoride varnish application increased from 3.6% (95% CI, 2.8%-4.4%) to 5.8% (95% CI, 4.5%-7.1%). Fluoride varnish applications were most common in Rhode Island, with a regression-adjusted probability of 8.7% (95% CI, 5.1%-12.4%). New Hampshire had a lower rate, with a regression-adjusted probability of 2.2% (95% CI, 1.2%-3.3%) of a visit including fluoride varnish.

**Table.** Characteristics of Well-Child Visits With and Without Fluoride Varnish Applications

Characteristic	Overall (N = 328 661)	Observation, No. (%)		Unadjusted odds of fluoride varnish application, OR (95% CI)^**a**^
		Visit with fluoride varnish application (N = 15 756)	Visit without fluoride varnish application (N = 312 905)	
Visit included fluoride varnish application	15 756 (4.8)	15 756 (100)	312 905 (0)	NA
Age, y				
1	132 563 (40.3)	7438 (47.2)	125 125 (40.0)	13.6 (8.1-22.9)^b^
2	70 365 (21.4)	5749 (36.5)	64 616 (20.7)	20.3 (13.8-30.0)^b^
3	46 345 (14.1)	2069 (13.1)	44 276 (14.2)	10.7 (7.3-15.5)^b^
4	39 693 (12.1)	327 (2.1)	39 366 (12.6)	1.9 (1.6-2.3)^b^
5	39 695 (12.1)	173 (1.1)	39 522 (12.6)	[Reference]
Sex				
Male	169 001 (51.4)	8129 (51.6)	160 872 (51.4)	[Reference]
Female	159 660 (48.6)	7627 (48.5)	152 033 (48.6)	1.0 (0.95-1.0)
Insurance type				
Preferred provider organization	171 273 (52.1)	8795 (55.8)	162 478 (51.9)	1.2 (0.9-1.7)
Health maintenance organization	84 049 (25.6)	3363 (21.3)	80 686 (25.8)	0.9 (0.7-1.4)
Point of service	68 702 (20.9)	3401 (21.6)	65 301 (20.9)	1.2 (0.9-1.6)
Exclusive provider organization	4637 (1.4)	197 (1.3)	4440 (1.4)	[Reference]
Year of visit				
2016	111 156 (33.8)	4060 (25.8)	107 096 (34.2)	[Reference]
2017	110 892 (33.7)	5563 (35.3)	105 329 (33.7)	1.4 (1.1-1.8)^b^
2018	106 613 (32.4)	6133 (38.9)	100 480 (32.1)	1.6 (1.3-2.1)^b^
State of residence				
Connecticut	134 662 (41.0)	3829 (24.3)	130 833 (41.8)	[Reference]
Maine	80 174 (24.4)	6410 (40.7)	73 764 (23.6)	3.0 (1.5-6.1)^b^
New Hampshire	64 584 (19.7)	1401 (8.9)	63 183 (20.2)	0.8 (0.3-1.8)
Rhode Island	49 241 (15.0)	4116 (26.1)	45 125 (14.4)	3.1 (1.3-7.2)^b^

Abbreviation: OR, odds ratio.

^a^ OR and 95% CIs from logistic regression models where the unit of observation was a well-child medical visit; models were estimated separately for each of the independent variables of interest and included county-level cluster robust standard errors.

^b^ Indicates difference in OR is statistically significantly different from the reference group.

**Figure.**
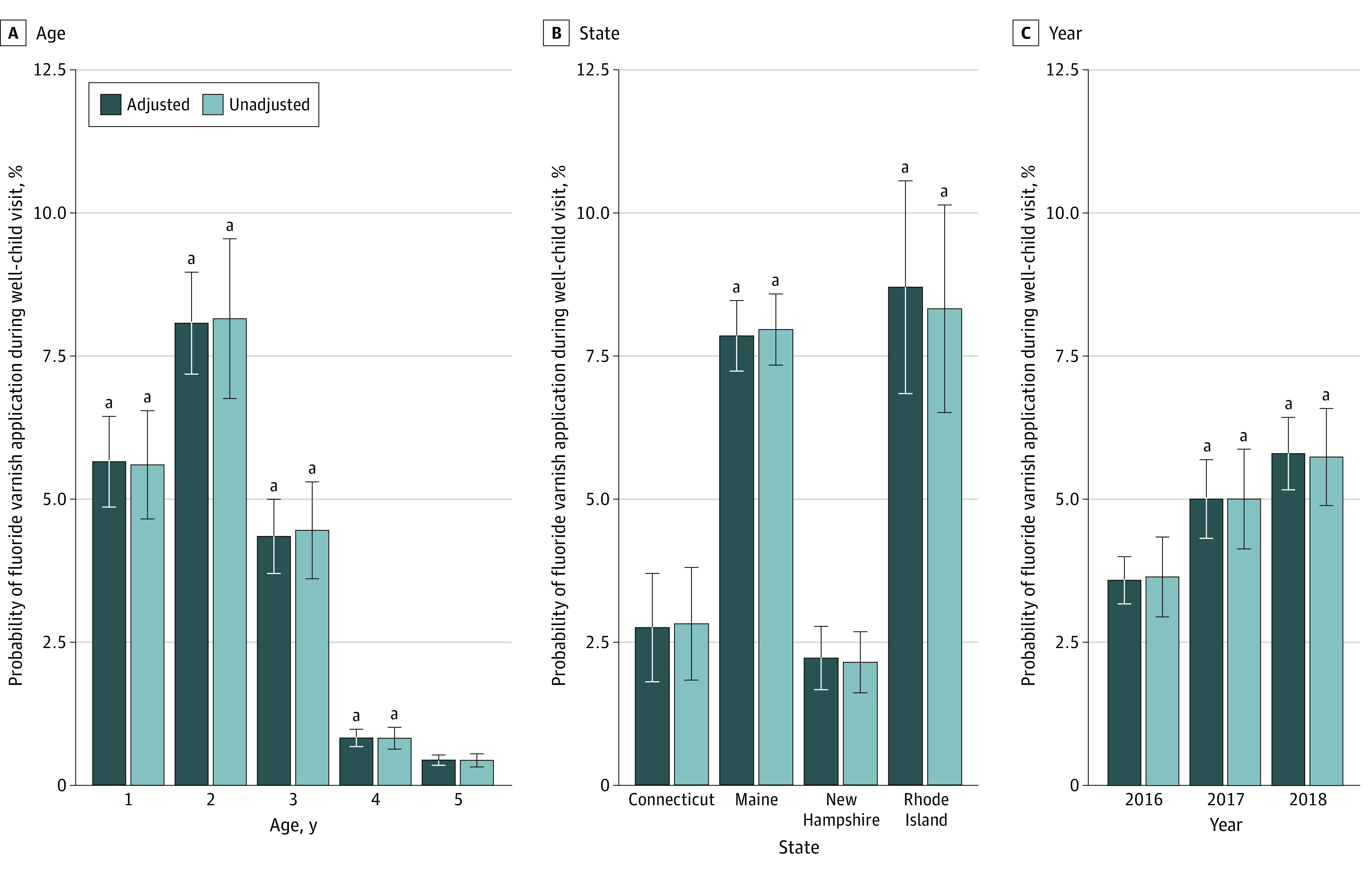
Regression-Adjusted Rates of Fluoride Varnish Applications During Well-Child Medical Visits, (N = 328 661) The figure presents unadjusted and regression-adjusted probabilities. Unadjusted probabilities are derived from logistic regression models predicting fluoride varnish applications during well-child medical visits. Models are estimated separately for each of the independent variables of interest (ie, age, state, and year). Regression-adjusted results are from a model controlling for child sex, age, insurance type, visit year, and state. In all models, standard errors are clustered at the county level, and the delta method was used to calculate standard errors for predicted probabilities. We calculated probabilities of fluoride varnish application during a well-child medical visit for each variable category of interest using model estimates to compute the mean of predicted probabilities for the entire sample after setting the covariate of interest (eg, Connecticut) while keeping all other covariates at their observed values. ^a^ Indicates difference in predicted probability is statistically significantly different from the reference group (child age = 5 years, state = Connecticut, year = 2016) at the 5% level.

## Discussion

Despite mandatory insurance coverage for fluoride varnish applications in medical settings, we found fewer than 5% of well-child visits for privately insured young children included this service, suggesting efforts are needed to increase pediatric medical providers’ delivery of fluoride varnish. Young children have few dental visits^6^ and delivering fluoride varnish in medical settings can increase access to preventive oral health services. Although this study was limited to 4 states, these data included a variety of private insurers and clinicians. Differences in fluoride varnish applications across states may be driven by variation in access to dentists and Medicaid payment policy. This study was the first to assess delivery of an evidence-based service for children who are privately insured, which is recommended by the US Preventive Service Task Force and the American Academy of Pediatrics. Although increases over time were encouraging, very low rates of fluoride varnish in medical settings suggest substantial expansion of this service in medical settings is critical for improving children’s oral health and overall well-being.

## Notes

Supplement.
eMethods.
