# Supplementary material for: Dental Fluoride Varnish Application During Medical Visits Among Children Who Are Privately Insured
Source: JAMA Netw Open. 2021 Aug 30;4(8):e2122953. doi: 10.1001/jamanetworkopen.2021.22953 (PMC8406076; doi:10.1001/jamanetworkopen.2021.22953)
Supplement: Supplement. — eMethods. [file jamanetwopen-e2122953-s001.pdf]

eSupplemental Online Content

Geissler KH, Dick AW, Goff SL, Whaley C, Kranz AM. Dental fluoride varnish application during medical visits among children who are privately insured. *JAMA Netw Open*. 2021;4(8):e2122953. doi:10.1001/jamanetworkopen.2021.22953

eMethods.

This supplemental material has been provided by the authors to give readers additional information about their work.

© 2021 Geissler KH et al. *JAMA Network Open.*

eMethods.

This cross-sectional study used 2016-2018 data for privately insured children from all payer claims databases from the Maine Health Data Organization, Connecticut Office of Health Strategy, New Hampshire Department of Health and Human Services, and State of Rhode Island Department of Health. We calculated descriptive statistics and estimated the unadjusted odds of a visit including fluoride varnish using logistic regression models. We also estimated a multivariable logistic regression model with the binary outcome of whether the visit included fluoride varnish application and independent variables of age, sex, insurance type, state, and visit year. Using the regression output, we then calculated regression-adjusted probabilities of fluoride application for age, state, and year. We used a significance level of 0.05 and county-level cluster robust standard errors. This clustering allowed for correlation in outcomes of children in the same county and accounted for multiple observations for the same child. Analyses used SAS Version 9.4 and Stata-MP version 16.1.

© 2021 Geissler KH et al. *JAMA Network Open.*
